# Allelic and phenotypic heterogeneity in Junctophillin-3 related neurodevelopmental and movement disorders

**DOI:** 10.1038/s41431-021-00866-1

**Published:** 2021-04-06

**Authors:** Thomas Bourinaris, Alkyoni Athanasiou, Stephanie Efthymiou, Sarah Wiethoff, Vincenzo Salpietro, Henry Houlden

**Affiliations:** 1 Department of Neuromuscular disorders, Institute of Neurology, University College London, London, UK; 2 Pediatric Neurology and Muscular Diseases Unit, IRCCS Giannina Gaslini Institute, Genoa, Italy; 3 Department of Neurosciences, Rehabilitation, Ophthalmology, Genetics, Maternal and Child Health, University of Genoa, Genoa, Italy

**Keywords:** Genetics research, Movement disorders

## Abstract

Junctophilin-3 belongs to a triprotein junctional complex implicated in the regulation of neuronal excitability and involved in the formation of junctional membrane structures between voltage-gated ion channels and endoplasmic (ryanodine) reticular receptors. A monoallelic trinucleotide repeat expansion located within the junctophilin-3 gene (*JPH3*) has been implicated in a rare autosomal dominant (AD) late-onset (and progressive) disorder clinically resembling Huntington disease (HD), and known as HD-like 2 (HDL2; MIM# 606438). Although the exact molecular mechanisms underlying HDL2 has not yet been fully elucidated, toxic gain-of-function of the aberrant transcript (containing the trinucleotide repeat) and loss of expression of (full-length) junctophilin-3 have both been implicated in HDL2 pathophysiology. In this study, we identified by whole exome sequencing (WES) a *JPH3* homozygous truncating variant [NM_020655.4: c.17405dup; p.(Val581Argfs*137)]. in a female individual affected with genetically undetermined neurodevelopmental anomalies (including delayed motor milestones, abnormal social communication, language difficulties and borderline cognitive impairment) and paroxysmal attacks of dystonia since her early infancy. Our study expands the *JPH3*-associated mutational spectrum and clinical phenotypes, implicating the loss of Junctophilin-3 in heterogeneous neurodevelopmental phenotypes and early-onset paroxysmal movement disorders.

## Introduction

Huntington’s disease (HD)-like 2 (HDL2) is a progressive autosomal dominant (AD) neurodegenerative disorder reported to be almost indistinguishable clinically from HD [1]. HDL2 typically presents in midlife initially with weight loss and diminished coordination and progresses to motor signs including dystonia, chorea, rigidity, bradykinesia, hyperreflexia, speech disturbances, and tremor [2]. Psychiatric symptoms (e.g., depression, anxiety, apathy, irritability and hallucinations) are also frequently observed [3]. The natural history of the disorder can be complicated by profound dementia and affected individuals can reach a non-verbal state and die within 10–20 years after disease onset due to severe neurological deterioration. HDL2 could be considered a very rare genetic disorder, estimated to occur in ~1% of patients clinically diagnosed with HD and negative for *HTT* variants. HDL2 individuals carry a CAG/CTG trinucleotide repeat (44–60 triplets) located within the *JPH3* gene (MIM# 605268) on chromosome 16q24. This gene encodes for junctophilin-3 (*JPH3*), a protein part/component of the junctional complex critically involved in the regulation of neuronal excitability and intracellular calcium signaling pathways at the endoplasmic reticulum (ER) [4–6]. Haplotype studies in HDL2 families from South Africa and North America provided some evidences for an African founder triplet expansion [6–8]. No heterozygous or biallelic variants in *JPH3* have been reported before. Recently, it has been suggested that de novo variants in ATN1, a gene associated with another repeat expansion neurodegenerative movement disorder (DRPLA), can cause a more complex neurodevelopmental phenotype [9]. In this study, we describe the phenotype of an individual affected with infantile onset paroxysmal dystonia as well as heterogeneous neurodevelopmental anomalies, found by whole exome sequencing (WES) to carry a homozygous truncating variant in the *JPH3* gene. We also discuss the heterogeneous clinical phenotypes and molecular mechanisms implicated in *JPH3*-related neurological disorders.

## Clinical report

**Fig. 1:**
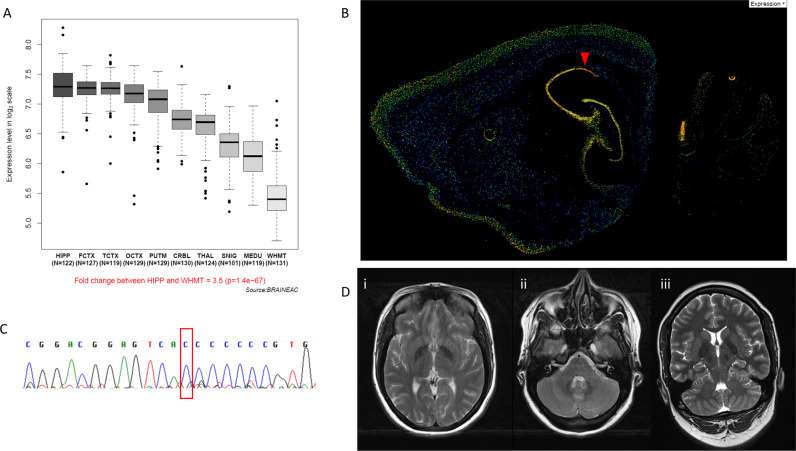
JPH3 expression, Sanger sequencing and imaging. **A** JPH3 is expressed in various brain regions, with the highest transcript. level in the hippocampus (from the BRAINEAC). HIPP, hippocampus; FCTX, frontal cortex; TCTX, temporal cortex; OCTX, occipital cortex; PUTM, putamen (at the level of the anterior commissure); CRBL, cerebellar cortex; THAL, thalamus SNIG, substantia nigra; MEDU, inferior olivary nucleus (sub-dissected from the medulla); WHMT, intralobular white matter (at the level of the lateral geniculate nucleus). **B** Panel from the Allen Reference Atlas, using annotated and colour-coded in situ hybridization data to show JPH3 expression in mouse tissue (from Mouse Brain Atlas) **C** Sanger sequencing electropherogram confirming the *JPH3* variant in the proband compared to the normal Sanger trace from a healthy control. **D** Brain MRI scan axial (i, ii) and coronal (iii) images showing normal findings, including no cerebral or cerebellar atrophy and no white matter lesions or basal ganglia abnormalities.

The index case is a 32-year-old female adopted at the age of 3 years (no detailed family information available) who was born 9 weeks preterm with no complications. Growth parameters and occipitofrontal circumference at birth were all within normal limits. A diagnosis of cerebral palsy was made in the first months of life and she was found to have delayed developmental milestones, including motor difficulties and abnormal social communication and language. Later on, she was diagnosed with learning difficulties and speech impairment. She started to walk autonomously at the age of 3 years. Since the second year of her life, she showed a movement disorder phenotype consisting of frequent episodes of generalized dystonic posturing affecting the trunk, neck and upper limbs bilaterally. During these episodes the girl experienced speech difficulties, facial flushing and, occasionally, muscle weakness resulting in frequent falls without loss of consciousness (Video 1). Attacks occurred on a daily basis during childhood with duration ranging between few seconds to several minutes. After adolescence the average duration has reduced from around 30 min to less than a minute. Reversely, frequency of episodes has increased from 1–2 to over 20 episodes. Triggers include emotional stress or extreme temperatures, although no clear association was reported. A number of medications, including lamotrigine, diazepam and levodopa, have not been successful in reducing frequency or severity of the attacks. In-between episodes, the patient is usually mobile and functional, although impaired by significant fatigue. On examination (Video 2), the patient is able to stand and walk without assistance. Gait is clumsy and she is not able to tandem walk. Range of eye movements is full with jerky pursuit and dysmetric saccades. Speech is slurred and dysarthric with reduced range of tongue movements. Power is mildly reduced in upper limbs distally, scoring 4/5 on the MRC scale. There is increased tone bilaterally and brisk deep tendon reflexes. Plantar reflexes are upgoing bilaterally confounded by withdrawal reaction. There is a bilateral fine rest, postural and intentional tremor, while cerebellar tests show significant dysmetria upon finger-nose- and finger-chase-tests bilaterally. There is also some generalized extrapyramidal slowing including reduced velocity of finger taps although no decrease in amplitude is observed. There are no hearing or visual problems reported and sphincter functions are reported normal apart from urinary urgency during some dystonic episodes. The patient reports intermittent pins and needles in both hands and feet in accordance with a potential clinical diagnosis of an early polyneuropathy, but no nerve conduction studies were available at the time of the report. Regular blood tests, lumbar puncture, EEG and MRI (Fig. 1D) were normal.

## Materials and methods

After institutional review board approval of this study and informed consent from the patient, we collected a blood sample and extracted DNA using standard procedures. To investigate the possible genetic cause of her disease, WES of the affected individual was performed. Nextera Rapid Capture Enrichment kit (Illumina, San Diego, California, USA) was used according to the manufacturer instructions. Libraries were sequenced on an Illumina HiSeq3000 using a 100 bp paired-end reads protocol. Sequence alignment to the human reference genome (UCSC hg19), and variants calling and annotation was performed as described elsewhere [10, 11] In total, 67,652,818 (II-1) unique reads were generated. In accordance with the phenotype, priority was given to low frequency variants [<1% in public databases, including 1000 Genomes project, NHLBI Exome Variant Server, Complete Genomics 69, and Exome Aggregation Consortium (ExAC v0.2)] that were located in genes previously associated with neurological and movement disorders phenotypes or that were fitting a recessive model of inheritance (i.e., homozygous or compound heterozygous).

## Results

Based on the bioinformatic analysis of the family WES data, we did not identify plausible compound heterozygous variants. A single rare homozygous variant was identified in *JPH3* (NM_020655.4), leading to a change in the gene reading frame [NM_020655.4: c.1740dup; p.(Val581Argfs*137)]. Thus, this variant emerged as the most likely explanation for the disease pathogenesis as supported by the previous implication of this gene in hyperkinetic movement disorders and the predicted severe disruption of a gene intolerant to loss-of-function (LoF; PLI = 0.99). The variant was absent from the GnomAD database (https://gnomad.broadinstitute.org/) where only three LoF (truncating) variants are reported in the *JPH3* gene [(p.(Trp81Ter), p.(Lys310Ter), p.(Ile741Thrfs*8)], all of them in the heterozygous state. Validation of WES results by Sanger sequencing confirmed the *JPH3* variant in the homozygous state in the affected individual (Fig. 1C) and was submitted to Leiden Open Variation Database (http://www.lovd.nl/JPH3; Individual ID: #0000697177). DNA samples from parents or relatives were not available as the patient is adopted with no contact to her biological family.

Consistent with the tissue-restricted expression pattern of the gene (https://www.proteinatlas.org/ENSG00000154118-JPH3/tissue) and its brain-specificity documented by previous studies [12], functional assays directed to analyze the impact of the *JPH3* homozygous truncating variant on transcripts and proteins (using patient-derived fibroblasts cell lines) failed to functionally evaluate the variant.

## Discussion

**Fig. 2:**
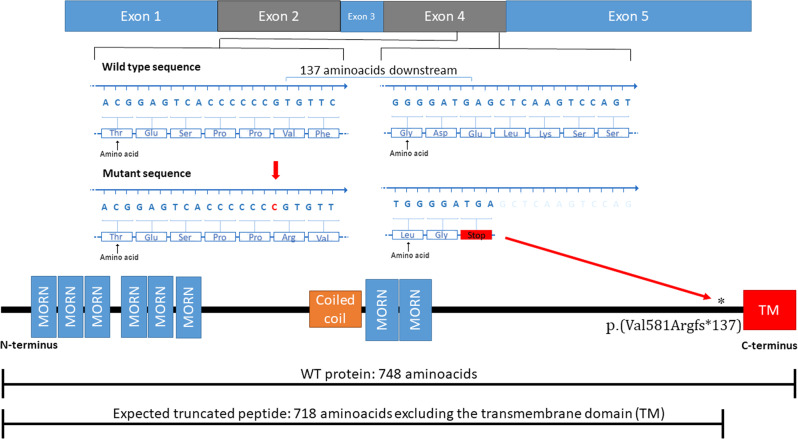
JPH3 Variant identified in this study.

HDL2 is a rare progressive and late-onset AD neurodegenerative disorder clinically resembling HD and caused by a monoallelic trinucleotide CTG/CAG repeat expansion located within the alternatively spliced exon 2A of *JPH3*. Overexpression of *JPH3* transcripts containing an expanded CUG repeat expansion have been shown to cause cellular toxicity, suggesting that the RNA transcripts play an important role in the HDL2 pathogenesis via a toxic RNA gain-of-function effect [13]; however, also loss of full-length *JPH3* protein expression has been potentially implicated in the disease pathogenesis [1]. So far, no individuals have been reported with biallelic variants in *JPH3*. In this study, we identified a homozygous c.1740dup variant within the fourth exon of the gene, affecting the canonical isoform (isoform 1: NP_065706.2) of *JPH3* and leading to the generation of a premature stop codon 137 amino acids downstream. The result is a putative Jph3 product of 718 amino acids, with a resulting function that is highly likely to be disrupted due to the absence of the downstream C-terminal transmembrane (TM) motif, which anchors the protein into the ER (Fig. 2). Importantly, the C-terminal TM domain of junctophilins is highly conserved across species and is known to play critical roles at the interface between intracellular membranes (e.g., ER/SR) and plasmalemma [14].

Our study implicates a disruptive *JPH3* LoF variant in heterogeneous neurodevelopmental issues and movement disorders with onset in the infantile age. According to GnomAD database containing WES data from 125,748 individuals (last accessed February 2021), *JPH3* is a gene highly intolerant to LoF (PLI = 0.99). Of note, several individuals have been reported in the Decipher database (https://decipher.sanger.ac.uk) carrying small 16q24 microdeletions encompassing the *JPH3* gene and presenting variable phenotypes of neurodevelopmental impairment, dysmorphic features and movement abnormalities [DECIPHER IDs: 413948, 280733, 326419].

Interestingly, a previous study identified by array-CGH a 16q24.1-16q24.2 deletion in a child affected with intellectual disability and seizures and the identified deletion encompassed in total four genes, of which *JPH3* had the highest LoF intolerance scores [15]. Furthermore, a recent genome-wide analysis established a significant association between a single nucleotide polymorphism in *JPH3* (rs1864152) and higher risks of autism spectrum disorders [16]. Based on the results identified in this study, we suggest that *JPH3* is a dosage-sensitive (haploinsufficient) disease gene and some allelic and phenotypic heterogeneity may occur in *JPH3*-related neurological disorders. Intriguingly, the delineation of the phenotypic spectrum associated with genetic defects of *JPH3*, including triplet expansions or (disruptive) intragenic variants, reveal a broad phenotypic spectrum including early developmental delay and (paroxysmal) dystonia as well as late cognitive impairment (often progressing to dementia) and different hyperkinetic movements. In the last decade, the advent of next generation sequencing technologies led to the identification of a number of pediatric monogenic diseases peculiarly characterized by paroxysmal/fluctuating episodes of dystonic posturing on a background of delayed developmental milestones. These include some dominant and recessive disorders caused by defects in pre- and post- synaptic genes (e.g., *PRRT2*, *ADCY5*, *PDE2A*, *PDE10A*) involved in the regulation of neuronal excitability (at central synapses) and/or implicated in brain c-AMP metabolism [17–20]. Based on the similar age of onset and clinical characteristics, these conditions should also be considered in the differential diagnosis of *JPH3*-related neurological disorders. In conclusion, our study expands both *JPH3*-related mutation mechanisms and clinical phenotypes. Last but not least, the confirmed implication of *JPH3* in broad neurodevelopmental anomalies, highlights an important role of this gene in human brain development and function, warranting further investigations.

## Supplementary information

Video 1

Video 2
